# Evaluation of maxillary anterior teeth and their relation to the golden proportion in malaysian population

**DOI:** 10.1186/1472-6831-13-9

**Published:** 2013-01-24

**Authors:** Maan Ibrahim Al-Marzok, Kais Raad Abdul Majeed, Ibrahim Khalil Ibrahim

**Affiliations:** 1 Department of Restorative Dentistry, MAHSA University College, Level 6, Block E, Pusat Bandar Damansara, 50490, Kuala Lumpur, Malaysia; 2 Department of Conservative Dentistry, International Islamic University Malaysia, Kuantan, Malaysia; 3 Department of Prosthetic Dentistry, Ajman University of Science and Technology, Ajman, United Arab Emirates

**Keywords:** Golden proportion, Malaysian ethnics, Aesthetics, Anterior teeth

## Abstract

**Background:**

The maxillary anterior teeth are important in achieving pleasing dental aesthetics. Various methods are used to measure the size and form of them, including the golden proportion between their perceived widths, and the width-to-height ratio, referred to as the golden standard. The purpose of this study was conducted to evaluate whether consistent relationships exist between tooth width and height of the clinical crown dimensions; and to investigate the occurrence of the golden proportion of the maxillary anterior teeth.

**Methods:**

Dental casts of the maxillary arches were made in this cross-sectional study from MAHSA University College students who met the inclusion criteria. The 49 participants represented the Malaysian population main ethnics. The dimensions of the anterior teeth and the perceived width of anterior teeth viewed from front were measured using a digital caliper.

**Results:**

Comparison of the perceived width ratio of lateral to central incisor and canine to lateral incisor with the golden proportion of 0.618 revealed there were a significant statistical difference (p < 0.05). The statistical difference was significant for the width-to-height ratio of central incisors to the golden standard of 80%. There was no significant difference in the comparison among ethnic groups for the golden proportion and the golden standard.

**Conclusions:**

The golden proportion was not found to exist between the perceived widths of maxillary anterior teeth. No golden standard were detected for the width-to-height proportions of maxillary incisors. Specific population characteristics and perception of beauty must be considered. However, ethnicity has no association with the proportions of maxillary anterior teeth.

## Background

Dental esthetics is a primary consideration for patients. New dental materials and techniques were introduced maximizing the likelihood of an attractive outcome. The size and form of the maxillary anterior teeth are important not only to dental esthetics, but also to facial esthetics [1].

A system of esthetic predictions is described by Levin. The application of this system is facilitated by the use of a dental grid for the anterior esthetic segment [2], its use may help simplify the diagnosis of facial and dental disharmonies and treatment using this system will help restore optimal facial esthetics [3].

Dental and facial aesthetics are optimized if proportion between widths of maxillary anterior teeth is repeated when the patient is viewed from the front [4]. This proportion is called golden proportion and is approximately 0.618. In this manner the visible width of lateral incisor is 62% (0.618) of central incisor and the visible width of canine is 62% (0.618) of lateral incisor [5]. This golden proportion has been proposed in many articles and textbooks as an esthetic guideline for restoring and replacing maxillary anterior teeth [6,7].

One of the most important guidelines is golden standard value. According to this standard, the ideal width-to-height proportion of maxillary central incisor should be approximately 80% width compared with height [8]. A higher width/height ratio means a squarer tooth, and a lower ratio indicates a longer appearance [9].

The objective of the present study was to investigate the occurrence of the golden proportion between the perceived widths of the maxillary anterior teeth. A second objective was to evaluate whether consistent relationships exist between tooth width and height of the clinical crown dimensions. The null hypotheses were that there is no difference between the proportions of maxillary anterior teeth of Malaysian population and the golden proportion or the golden standard values. The study also aimed to compare these proportions among the 3 majority ethnic groups in Malaysia: Malay, Chinese and Indian.

## Methods

A cross-sectional study was conducted over a period of 4 months for students of MAHSA University College in Pusat Bandar Damansara Campus.

The participants are selected according to the following criteria:

1. Complete maxillary and mandibular anterior teeth.

2. No periodontal disease.

3. No spacing and crowding in anterior maxillary teeth.

4. No history of orthodontic treatments.

5. No intruded, extruded or rotated teeth in the anterior region.

Using these criteria, 49 students were selected for evaluation represented the Malaysian population main ethnics: (22 Chinese, 14 Indians and 13 Malay). Their mean age was 18–23 years. Participants were asked to identify their ethnicity by selecting an answer using pre-fixed ethnicity categories (e.g. Malay, Chinese, Indian, Indigenous people, Mixed and Others). Then participants were also asked to identify their father’s and mother’s ethnicity using the same pre-fixed categories.

Ethical approval obtained from the Ethics Committee of the Faculty of Dentistry, MAHSA University College. All volunteers participated in the research signed informed consent prior to their participation which included the nature of the project and declared the confidentiality of all information.

**Figure 1:**
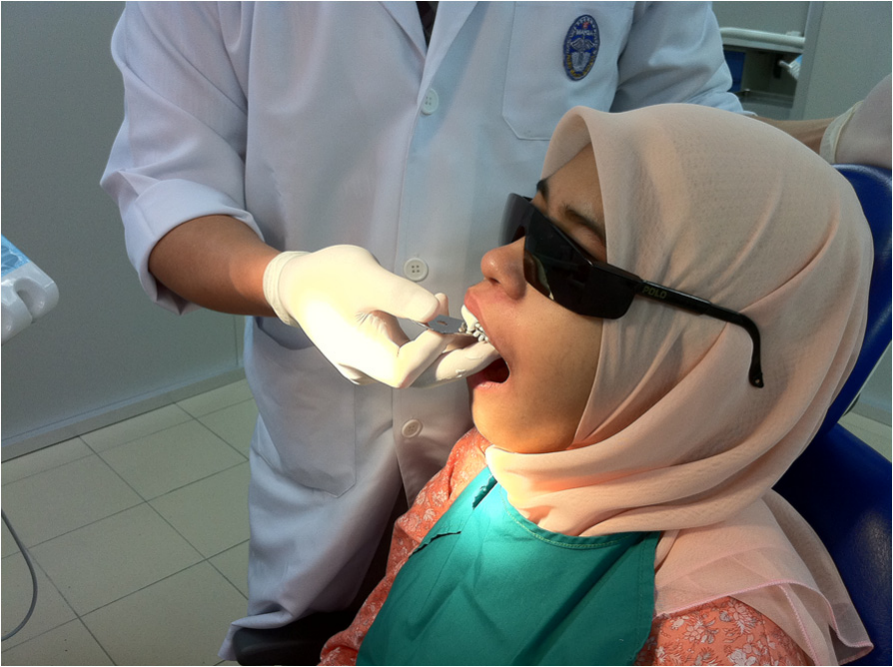
Making an alginate impression for maxillary arch of a volunteer.

Irreversible hydrocolloid impressions of the maxillary arches were made in stock trays and poured with Type IV dental stone. Making an alginate impression for the maxillary arch of a volunteer is shown in (Figure 1).

**Figure 2:**
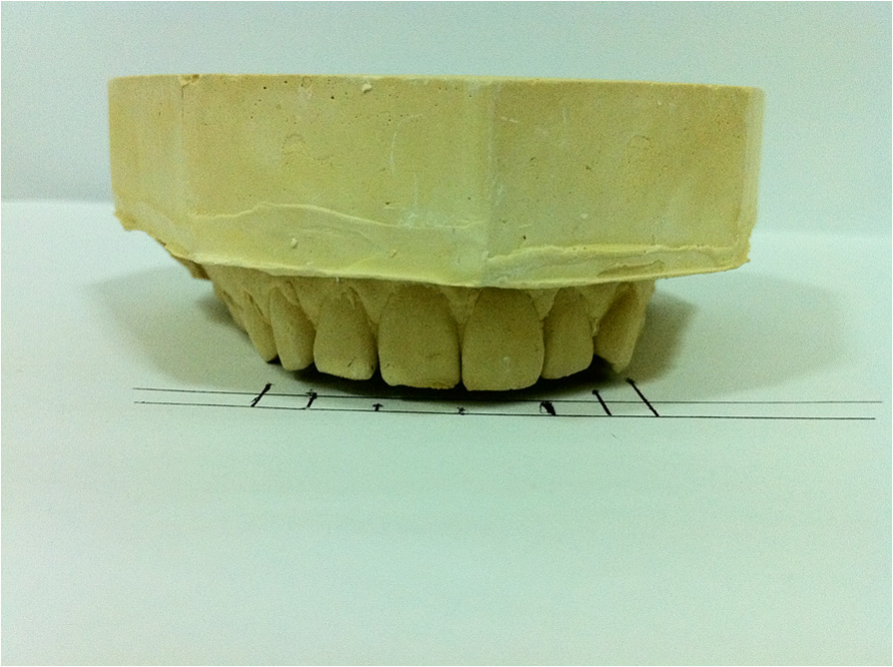
Drawing of the grids for the perceived widths of maxillary anterior teeth.

The dimensions of the anterior teeth and the perceived width of anterior teeth viewed from front were measured using a digital caliper read to the nearest 0.01 mm. Evaluations regarding the occurrence of the golden proportion were conducted by drawing of grids that obtained by placing the casts on a flat surface and drawing vertical lines representing the perceived mesiodistal widths of the teeth. The golden proportion grid is shown in (Figure 2).

**Figure 3:**
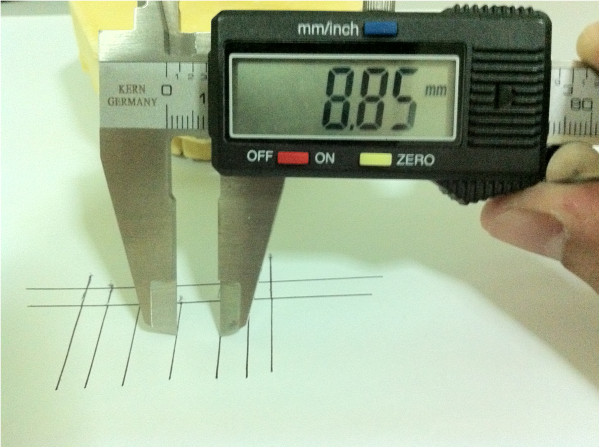
Taking measurements for the spaces in the golden proportion grid.

Measurements were done for the spaces in the grids using the digital caliper as shown in (Figure 3). All measurements were performed by the three researchers working independently and the average of these measurements was taken; if the readings differed by more than 0.2 mm, the procedure was repeated.

The 3 sets of measurements of each researcher were compared to check the intra-examiner repeatability. Intraclass correlation coefficient was 0.998, 0.996 and 0.988. Inter-examiner repeatability checked among the researchers and the correlation coefficient was 0.991. These results showed that the measurements could be repeated with high accuracy.

A one-sample *t* test was used to compare the width-to-height ratios of all tooth groups with the proportion of 80% and to assess the incidence of the golden proportion of 0.618. One-way analysis of variance (ANOVA) was used to analyse the comparison among ethnics in the golden proportion and golden standards.

Statistical analyses were carried out using Statistical Package for Social Sciences (SPSS 16.0) with the *Level of Significance* α = 0.05 and *Degree of confidence* d = 0.95.

Post hoc power analysis was made using PS power and sample size program as suggested by Dupont and Plummer (1998) [10].

## Results

The occurrence of golden proportion within the range of 0.55 to 0.64 (as measured by Mahshid et al., 2004) was found in 20.4% of the perceived lateral-to-central incisor ratios and in 20.4% of the perceived canine-to-lateral incisor ratio. The existence of golden proportion of the perceived lateral-to-central incisor ratio among ethnicities were as follows: 13.6% of Chinese, 35.7% of Indians and 15.4% of Malay. The existence of golden proportion of the perceived canine-to-lateral incisor ratio among ethnicities were as follows: 13.6% of Chinese, 21.4% of Indians and 30.8% of Malay.

**Figure 4:**
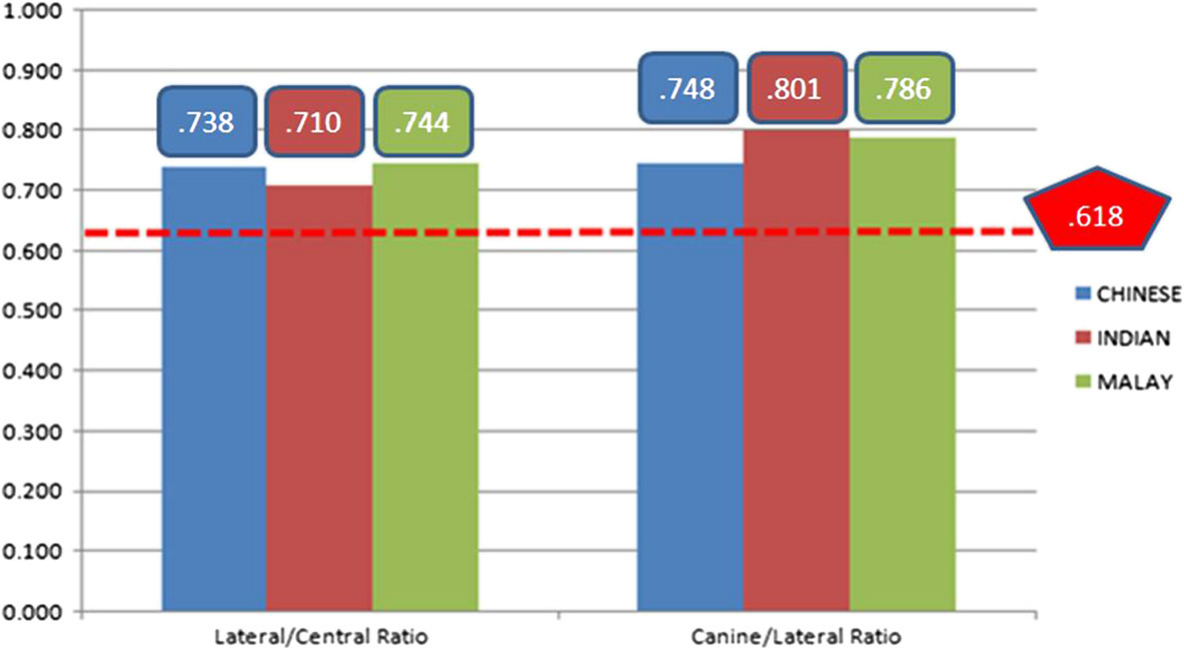
**Mean values of the ratio of perceived widths of lateral to central incisor and canine to lateral incisor by ethnicity.** The dotted line, the golden proportion of 0.62.

Ratios for maxillary lateral to central incisors and canine to lateral incisor based on the golden proportion are shown in (Figure 4). Narrower central incisors and wider canines compared to the lateral incisors were found for all ethnic groups rather than the golden proportion.

One-way ANOVA was used to analyze the comparison between ethnics of the lateral to central incisor ratio is demonstrated in Table 1; and of the canine to lateral incisor ratio is demonstrated in Table 2. The ANOVA Tables showed there were no significant difference in the comparison among the ethnic groups for the golden proportion.

**Table 1:** ANOVA table for lateral/central incisor ratio in each ethnic group

**Ethnicity**	**N**	**Mean**	**SD**	**F- Statistic (df)**	**P value**
CHINESE	22	0.738	0.080	1.251 (2)	0.291 (Non-Significant)
INDIAN	14	0.710	0.083		
MALAY	13	0.744	0.104		

**Table 2:** ANOVA table for canine/lateral incisor ratio in each ethnic group

**Ethnicity**	**N**	**Mean**	**SD**	**F- Statistic (df)**	**P value**
CHINESE	22	0.748	0.092	1.949(2)	0.148 (Non-Significant)
INDIAN	14	0.801	0.139		
MALAY	13	0.786	0.133		

One-sample *t*-test statistics revealed there were significant differences (P < 0.05) emerged when the mean ratios between perceived widths of maxillary anterior teeth compared with the ideal golden proportion of 0.618 (P = 0.026 for lateral to central incisor ratio; P = 0.017 for canine to lateral incisor ratio). This indicates the golden proportion did not exist.

The occurrence of golden standard for the width-to-height ratio of maxillary central incisor within the range of 75% to 80% (as suggested by Peixoto et al., 2012) was found in 20.4% of the population. The existence of golden standard among ethnicities were as follows: 22.7% of Chinese, 21.4% of Indians and 15.4% of Malay.

**Figure 5:**
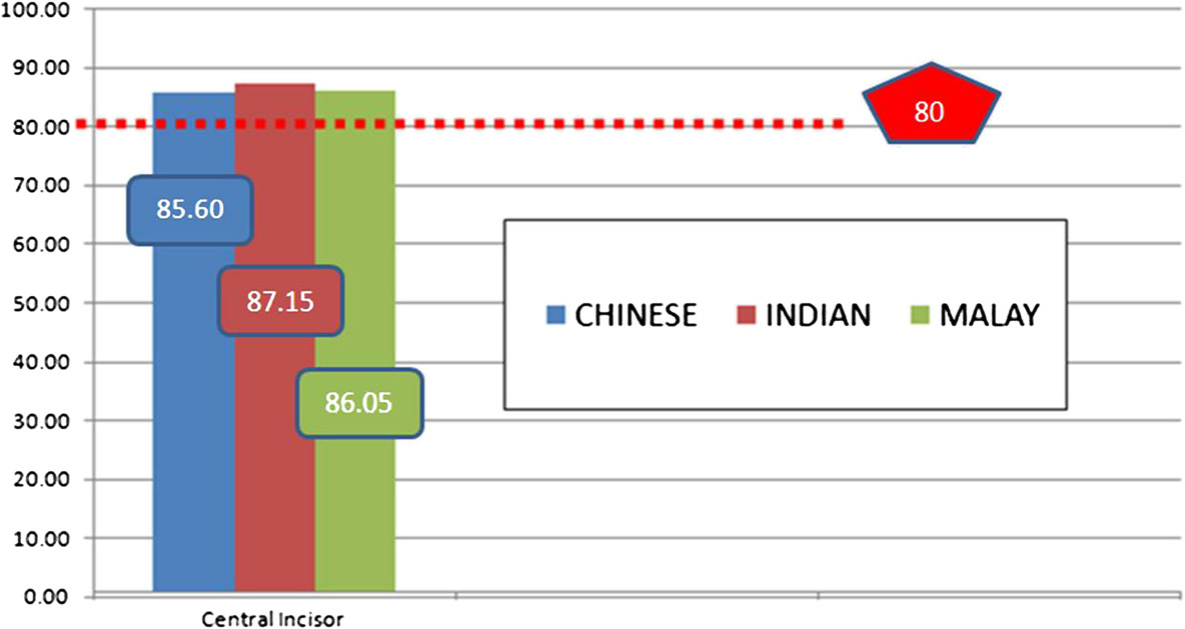
**Mean values (%) of the width-to-height ratio of maxillary central incisor.** The dotted line, the golden standard of 80%.

The means for the widths and heights of the maxillary central incisors are shown in (Figure 5). More square central incisors were found (more than 85% for all ethnics) for all ethnic groups rather than the golden standard.

Table 3 represents the one-way ANOVA that was used to analyze the comparison between ethnics of the width-to-height ratio of maxillary incisors. The ANOVA Table showed there was no significant difference among the ethnic groups for the golden standard.

**Table 3:** ANOVA table for width/height ratio of central incisor in each ethnic group

**Ethnicity**	**N**	**Mean (%)**	**SD (%)**	**F- Statistic (df)**	**P value**
CHINESE	22	85.60	5.28	0.471 (2)	0.625 (Non-Significant)
INDIAN	14	87.15	7.96		
MALAY	13	86.05	6.49		

One-sample *t*-test statistics was used for the assessment of width-to-height ratio of central incisors. There were significant differences (P = 0.022 < 0.05) emerged when the mean ratios compared with the proportion of 80% to assess the incidence of golden standards. This showed that no golden standard was observed.

## Discussion

The results of this study displayed no specific effect of ethnicity on golden proportion and golden standard among the three major Malaysian ethnic groups. Ethnicity has a close association with genetics. It is of no doubt that the ethnicity will affect the tooth proportions greatly between population and ethnics. However, this is an exception if the population has intermarried and not considered “pure” ethnic. Similar traits may be observed in different populations originate from same continent [11].

Many dental and facial characteristics differ following the geographical location and historical background. Therefore, information regarding tooth norms in a group of population is useful to dentists when restoring teeth [12]. The general Malaysian data can be used in the current study to compare with other populations as the golden proportion and golden standard was not found in all ethnic groups.

Determination of a mathematical or geometrical relation between anterior teeth is important to achieve an esthetic result. It would be helpful if statistically reliable results existed to support existing theories [13]. However, the golden proportion idea can no longer be considered since many articles found that golden proportion didn’t exist.

Rosenstiel and others found that golden proportion was preferred only when viewing very tall teeth and less desirable for normal height or shorter teeth [14]. Ward in 2001 recommended using other ratios, such as 0.70 rather than 0.618 to provide more pleasing appearance [15].

The current study found poor correlation between teeth dimensions and the golden proportion which is similar to `the findings of (Preston in 1993, Gillen et al. in 1994, Mahshid et al. in 2004, Hasanresioglu et al. in 2005, Fayyad et al. in 2006, Murthi and Ramani in 2008 and Petricevic et al. in 2008) [1,13,16-20].

The results for Malaysian population were comparable to the results reported in similar studies of other populations, including Turkish [1], Iranians [5,18], Jordanians [13], Americans [16], Indians [19] and Caucasians [20].

Peixoto et al. reported that the ideal W/H ratio for the central incisor should lie between 75 and 80%. However, the ratio which allows an aesthetically acceptable appearance is in the 65 to 85% range [9].

According to Hasanresioglu et al., the highest W/H ratio is found in squarer teeth due to shorter height and/or greater width than those of other population which came in agreement with the result of this study [1].

The results of this study showed W/H ratio higher than other studies (Hasanresioglu et al. in 2005, Wolfart et al. in 2006, Parnia et al. in 2010) although these studies estimated that there is no golden standard in the nature [1,5,8]. Recent study conducted in Korea showed similar results for the non-celebrities group [21]. These results might be attributed to differences in racial characteristics.

Power analysis was used to find how much power for this cross-sectional study if we had a specified number of volunteers. The power analysis of 65%. This indicates that there is a 65% chance of rejecting the null hypothesis when it’s false while 80% is generally considered to be good power.

With small sample size, the sample mean tends to be noticeably larger than when the null hypothesis is rejected with the larger sample size. Literatures reveal small difference in means between current study and studies with larger sample sizes.

In the present study, limitation such as minor inaccuracies common to the making of dental cast might have affected the measurements. Time constraints and the exclusion criteria restricted the number of volunteers who could be recruited into the study. Additional research on a greater sample size selected more systematically is needed before extrapolating the results to the Malaysian population.

## Conclusions

Within the limitations of this study:

1. The golden proportion was not found to exist between the perceived widths of maxillary anterior teeth.

2. No golden standard were detected for the width-to-height proportions of maxillary incisors.

3. Ethnicity has no association with the proportions of maxillary anterior teeth.

4. Specific population characteristics and perception of beauty must be considered.

## Competing interests

The authors declare that they have no competing interests.

## Authors’ contributions

MAL-M took part in making the study conception and design, supervision of the research group, analysis and interpretation of data, involved in drafting the manuscript. KRA-M participated in the design of the study and involved in drafting the manuscript. IKI involved in drafting the manuscript. Authors read and approved the final manuscript.

## Pre-publication history

The pre-publication history for this paper can be accessed here:

http://www.biomedcentral.com/1472-6831/13/9/prepub
